# A comparison of gene expression profiles in patients with coronary artery disease, type 2 diabetes, and their coexisting conditions

**DOI:** 10.1186/s13000-017-0630-7

**Published:** 2017-06-08

**Authors:** Rui Gong, Menghui Chen, Cuizhao Zhang, Manli Chen, Haibin Li

**Affiliations:** 1 Department of gerontology, The Third Municipal Hospital of Shijiazhuang City, Shijiazhuang, Hebei province 050011 China; 2 Department of cardiothoracic surgery, The Third Municipal Hospital of Shijiazhuang City, Shijiazhuang, Hebei province 050011 China; 3 Medical laboratory technology, The Third Municipal Hospital of Shijiazhuang City, Shijiazhuang, Hebei province 050011 China; 4 Department of Cardiology, The Third Affiliated Hospital of Hebei Medical University, Shijiazhuang, Hebei province 050051 China

**Keywords:** Type 2 diabetes, Coronary artery disease, Gene expression profile

## Abstract

**Background:**

To support a hypothesis that there is an intrinsic interplay between coronary artery disease (CAD) and type 2 diabetes (T2D), we used RNA-seq to identify unique gene expression signatures of CAD, T2D, and coexisting conditions.

**Methods:**

After transcriptome sequencing, differential expression analysis was performed between each disordered state and normal control group. By comparing gene expression profiles of CAD, T2D, and coexisting conditions, common and specific patterns of each disordered state were displayed. To verify the specific gene expression patterns of CAD or T2D, the gene expression data of GSE23561 was extracted.

**Results:**

A strong overlap of 191 genes across CAD, T2D and coexisting conditions, were mainly involved in a viral infectious cycle, anti-apoptosis, endocrine pancreas development, innate immune response, and blood coagulation. In T2D-specific PPI networks involving 64 genes, TCF7L2 (Degree = 169) was identified as a key gene in T2D development, while in CAD-specific PPI networks involving 64 genes, HIF1A (Degree = 124), SMAD1 (Degree = 112) and SKIL (Degree = 94) were identified as key genes in the CAD development. Interestingly, with the provided expression data from GSE23561, the three genes were all up-regulated in CAD, and SMAD1 and SKIL were specifically differentially expressed in CAD, while HIF1A was differentially expressed in both CAD and T2D, but with opposite trends.

**Conclusions:**

This study provides some evidences in transcript level to uncover the association of T2D, CAD and coexisting conditions, and may provide novel drug targets and biomarkers for these diseases.

**Electronic supplementary material:**

The online version of this article (doi:10.1186/s13000-017-0630-7) contains supplementary material, which is available to authorized users.

## Background

Type 2 diabetes (T2D) and coronary artery disease (CAD) often coexist and cause substantial public health and economic burden world-wide. CAD has long been established as a complication of T2D. The plaque formation in T2D patients may narrow the coronary arteries and thus predispose the occurrence of heart attack. It is assumed that there is an intrinsic interplay between T2D and CAD, in the form of shared etiology and pathophysiological mechanisms. The two diseases have shared common risk factors such as, age, gender, anthropometric, metabolic, socioeconomic and lifestyle variables, as well as psychosocial stress and environmental pollutant exposure. In addition, both diseases are characterized by a chronic inflammatory process [1, 2] and disorders of the coagulation system [3].

T2D has been associated with increased risk of cardiovascular disease and death [4, 5]. The underlying mechanisms may involve a complex interplay between genes predisposing to insulin resistance and those independently regulating lipid metabolism, coagulation processes and biological responses of the arterial wall [6]. The shared susceptibility regions (bin 9.3 and 6.5) were observed across T2D, obesity and CAD by Wu et al, suggesting the possibility of shared pathophysiology and risk through genetic pleiotropy [7], which may account for their frequent coexistence. However, there has been limited success in correlating T2D with CAD in terms of pathophysiologic changes up to now [8].

Peripheral blood gene expression profile has been used to reflect pathological conditions in a variety of diseases [9–14]. The increasing genomic information has provided an opportunity to better understand the complex biological processes of diseases, especially after the emergence of high throughput technologies. Previous studies have reported the gradual change in circulating gene expression profiles in patients with different extent of CAD [14].

Therefore, to support a hypothesis of an intrinsic interplay between T2D and CAD, we compared the peripheral blood gene expression profiles of T2D, CAD and coexisting conditions, to better understand the association of the three metabolic disorders.

## Methods

### Patients

All patients (2 T2D, 2 CAD, and 6 T2D + CAD) were recruited from the Third Municipal Hospital of Shijiazhuang City between March 2007 and December 2009. The diagnosis of T2D was according to World Health Organization criteria [15]. CAD was diagnosed with imaging techniques to detect flow-limiting coronary artery stenosis[16]. Patients met both of the above inclusion criteria were defined as T2D + CAD. The age- and race-matched patients (*n* = 7) attending the outpatient department were recruited as control during the study period. None of these patients had previous diagnosis of dyslipidemia, abnormal glucose tolerance, high blood pressure, or any illness. Demographic data and medication of the study population are summarized in Additional file 1: Table S1. The study was approved by the Institutional Review Board of the Third Municipal Hospital of Shijiazhuang City and all subjects provided written informed consent.

### RNA isolation and sequencing

Peripheral blood mononuclear cells (PBMCs) were isolated from ethylene diamine tetraacetic acid (EDTA) anticoagulated whole blood using Ficoll–Hypaque gradients. Total RNA was extracted using a Trizol reagent (Invitrogen, Carlsbad, CA, USA). The quality and quantity of RNA were evaluated on a Nanodrop ND-2000 spectrophotometer (Thermo Scientific, Wilmington, DE, USA). Isolation of messenger RNA (mRNA) was carried out using a TruSeq RNA library preparation kit (Illumina, San Diego, CA) according to the manufacturer’s instruction. The products were subsequently fragmented into sizes of around 200 bp and subjected to double-stranded cDNA synthesis. A HiSeqTM 2500 platform (Illumina) was applied to perform sequencing.

### Differential expression analysis

TopHat v1.3.1 software [17] was used to align raw sequencing reads to the UCSC human reference genome (Build hg19). The original alignment file was processed to measure transcript abundance using Cufflinks v1.0.3 software [18]. Transcript abundance of each gene was determined by calculation of Reads per kilobase of exon per million mapped reads (RPKM). The paired t-tests were performed to identify differentially expressed genes. *P* <0.05 was selected as the criteria for significant differences. Hierarchical clustering of differentially expressed genes was performed using the “pheatmap” function of the R/Bioconductor package [8].

### Functional enrichment analysis of differentially expressed genes

Gene ontology (GO) enrichment analysis and Kyoto Encyclopedia of Genes and Genomes (KEGG) pathway analysis were performed to annotate the biological function of the differentially expressed genes using the online software GENECODIS [19]. A cut-off of FDR was defined at 0.05.

### Protein-protein interactions (PPIs) network construction

To reveal the interactions of selected genes at molecular level, PPIs network was established based on the online database [20, 21]. Biological General Repository for Interaction Datasets (BioGRID) (http://thebiogrid.org/) was used to construct PPI networks, and the distribution characteristics of selected genes in the PPI network were visualized using Cytoscape software [22]. Nodes in the PPI network represent proteins, while edges represent interactions between two proteins.

### Verification of gene expression via GSE23561

The publicly available microarray dataset, GSE23561, was downloaded from GEO database (GEO, http://www.ncbi.nlm.nih.gov/geo) [23] to confirm the selected differentially expressed genes between each disorder group and normal control. In GSE23561, it showed the peripheral blood gene expression profiles of control (*n* = 9), rheumatoid arthritis (*n* = 6), metabolic syndrome (*n* = 6), CAD (*n*= 6) and T2D (*n* = 8).

## Results

### Analysis of transcriptome sequencing

A total of 3.28 × 10^7^, 2.88 × 10^7^, 3.30 × 10^7^, and 2.28 × 10^7^ sequencing reads were generated from the CAD, T2D, T2D + CAD, and control groups. Total number of reads that uniquely aligned to the UCSC human reference genome (hg.19) were 2.17 × 10^7^, 1.90 × 10^7^, 2.18 × 10^7^, and 1.51 × 10^7^, respectively.

### Differential expression analysis between T2D and control group

Four-hundred seventy four genes were identified to be significantly differentially expressed in T2D compared to the control group, including 126 up-regulated and 348 down-regulated genes. GO enrichment analysis and KEGG pathway analysis showed that 92 GO terms were significantly enriched, including G1/S transition of mitotic cell cycle (GO:0000082, P = 1.08E-05), transport(GO:0006810, P = 2.81E-05), etc. Blood coagulation (GO:0007596, P = 0.003), inflammatory response (GO:0006954, P = 0.007), endocrine pancreas development (GO:0031018, P = 0.002), and viral reproduction (GO:0016032,9.01E-06). Interestingly, pathway of Parkinson’s disease was identified as the highly significantly enriched pathway in T2D (P = 0.001), confirming that T2D may facilitate the development of Parkinson’s disease (Table 1).

**Table 1:** The top 15 significantly enriched pathways for differentially expressed genes in T2D

KEGG ID	KEGG term	Count	FDR	Genes
hsa05012	Parkinson's disease	10	1.33E-03	NDUFA5,ATP5J,NDUFV2,SNCA,NDUFA12,LRRK2,ATP5C1,UQCRB,VDAC3,UBB
hsa03010	Ribosome	8	1.41E-03	RPS3A,UBA52,RSL24D1,RPL7,RPL39,RPL21,RPS24,RPS12
hsa05200	Pathways in cancer	13	1.26E-02	PTCH1,FOS,BCL2L1,COL4A2,PTEN,BIRC3,TCF7,TCEB1,IL8,SMAD3,ITGB1,STK36,FN1
hsa05222	Small cell lung cancer	6	1.48E-02	BCL2L1,COL4A2,PTEN,BIRC3,ITGB1,FN1
hsa05016	Huntington’s disease	9	1.49E-02	NDUFA5,ATP5J,NDUFV2,NDUFA12,CREB5,ATP5C1,UQCRB,SP1,VDAC3
hsa04210	Apoptosis	6	1.50E-02	BCL2L1,BIRC3,IRAK3,DFFB,IL3RA,PRKACB
hsa05160	Hepatitis C	8	1.53E-02	STAT2,TBK1,IL8,MAVS,OAS3,OAS1,NR1H3,EIF3E
hsa04640	Hematopoietic cell lineage	6	1.56E-02	CD55,IL1R2,IL6R,TFRC,CD5,IL3RA
hsa04510	Focal adhesion	10	1.57E-02	COL4A2,PTEN,MYL12B,BIRC3,RAP1B,ARHGAP5,ITGB1,MYL12A,FN1,PPP1CC
hsa04114	Oocyte meiosis	7	1.67E-02	PPP2R5A,CDC27,SKP1,SLK,SMC3,PRKACB,PPP1CC
hsa04110	Cell cycle	7	1.76E-02	ORC3,CDC27,ORC4,SKP1,DBF4,SMAD3,SMC3
hsa03050	Proteasome	4	2.40E-02	PSMC6,POMP,PSMD12,PSMA4
hsa04120	Ubiquitin mediated proteolysis	7	2.54E-02	CDC34,CDC27,BIRC3,UBE2W,TCEB1,SKP1,CUL3
hsa04622	RIG-I-like receptor signaling pathway	5	2.56E-02	TBK1,ATG5,TANK,IL8,MAVS
hsa03013	RNA transport	7	3.28E-02	RANBP2,SRRM1,THOC7,NUP54,XPO1,POM121C,EIF3E

### Differential expression analysis between CAD and control group

A total of 488 genes were significantly differentially expressed in CAD when compared with the control group, which include 400 up-regulated and 88 down-regulated genes. Signal transduction (GO: 0007165, P = 6.14E-11) and blood coagulation (GO:0007596, P = 8.96E-09) were significantly enriched. Chemokine signaling pathway was one of the most significantly enriched pathways (P = 6.14E-11)(Table 2).

**Table 2:** The top 15 significantly enriched pathways for differentially expressed genes in CAD

KEGG ID	KEGG term	Count	FDR	Genes
hsa04062	Chemokine signaling pathway	15	8.37E-06	STAT2,CCL3L3,NRAS,RAP1A,CXCL1,RAP1B,CCL3,GNAI3,IL8,ADCY4,PIK3R1,PTK2,CCL4,JAK2,PRKACB
hsa05144	Malaria	8	2.26E-05	IL1B,IL6,HBD,SDC4,GYPC,THBS1,IL8,ICAM1
hsa04670	Leukocyte transendothelial migration	10	2.07E-04	MLLT4,RAP1A,RAP1B,GNAI3,ARHGAP5,ICAM1,PIK3R1,PTK2,ITGB1,MYL12A
hsa04621	NOD-like receptor signaling pathway	7	4.20E-04	IL1B,NLRP1,IL6,TNFAIP3,CXCL1,BIRC3,IL8
hsa05142	Chagas disease (American trypanosomiasis)	9	4.85E-04	IL1B,IL6,CCL3L3,PPP2R2A,CCL3,GNAI3,IL8,IFNGR1,PIK3R1
hsa05323	Rheumatoid arthritis	8	4.86E-04	IL1B,IL6,ATP6V1C1,CCL3L3,CXCL1,CCL3,IL8,ICAM1
hsa04010	MAPK signaling pathway	14	4.97E-04	IL1B,NRAS,RAP1A,RASA2,PPM1A,LAMTOR3,RAP1B,PLA2G6,DUSP5,NR4A1,CACNB3,DUSP2,PRKACB,MAP3K8
hsa03010	Ribosome	8	5.05E-04	RPS3A,UBA52,RSL24D1,RPL7,RPL39,RPL21,RPS24,RPS12
hsa05146	Amoebiasis	8	1.26E-03	IL1B,IL6,CXCL1,SERPINB2,IL8,PIK3R1,PTK2,PRKACB
hsa04510	Focal adhesion	11	1.31E-03	PTEN,RAP1A,THBS1,BIRC3,RAP1B,ITGA5,ARHGAP5,PIK3R1,PTK2,ITGB1,MYL12A
hsa05162	Measles	9	1.33E-03	IL1B,IL6,STAT2,TBK1,TNFAIP3,IFNGR1,PIK3R1,JAK2,OAS1
hsa04620	Toll-like receptor signaling pathway	8	1.43E-03	IL1B,IL6,TBK1,CCL3,IL8,PIK3R1,CCL4,MAP3K8
hsa05222	Small cell lung cancer	7	2.02E-03	BCL2L1,PTEN,BIRC3,PIK3R1,PTK2,ITGB1,PTGS2
hsa04210	Apoptosis	7	2.17E-03	IL1B,BCL2L1,BIRC3,PIK3R1,IRAK3,PRKACB,ATM
hsa05145	Toxoplasmosis	8	3.37E-03	BCL2L1,BIRC3,PLA2G6,GNAI3,IFNGR1,PIK3R1,ITGB1,JAK2

### Differential expression analysis between T2D + CAD and control group

For T2D + CAD, 370 genes were up-regulated and 69 genes were down-regulated. GO terms identified as most significantly enriched were signal transduction (GO: 0007165, P = 2.94E-07) and anti-apoptosis (GO: 0006916, P = 2.86E-06). Malaria was the most significantly enriched pathway (P = 0.00015) (Table 3).

**Table 3:** Highly significantly enriched pathways for differentially expressed genes in T2D + CAD group

KEGG ID	KEGG term	Count	FDR	Genes
hsa05144	Malaria	7	1.51E-04	IL1B,IL6,HBD,GYPC,IL8,HBA2,KLRB1
hsa03010	Ribosome	9	1.82E-04	RPS3A,UBA52,RSL24D1,RPL7,RPL39,RPL21,RPL34,RPS24,RPS12
hsa04620	Toll-like receptor signaling pathway	7	1.01E-02	IL1B,IL6,TBK1,CCL3,IL8,TLR5,CCL4
hsa05211	Renal cell carcinoma	6	1.18E-02	HIF1A,NRAS,RAP1A,PAK6,RAP1B,SLC2A1
hsa05020	Prion diseases	4	1.38E-02	IL1B,IL6,EGR1,PRKACB
hsa05143	African trypanosomiasis	4	1.38E-02	IL1B,IL6,HBD,HBA2
hsa04621	NOD-like receptor signaling pathway	5	1.54E-02	IL1B,IL6,CXCL1,BIRC3,IL8
hsa05323	Rheumatoid arthritis	6	1.59E-02	IL1B,IL6,CXCL1,ATP6V0E1,CCL3,IL8
hsa04062	Chemokine signaling pathway	9	1.64E-02	NRAS,RAP1A,CXCL1,RAP1B,CCL3,IL8,CCL4,JAK2,PRKACB
hsa04010	MAPK signaling pathway	10	1.94E-02	IL1B,NRAS,RAP1A,RASA2,PPM1A,LAMTOR3,RAP1B,NR4A1,DUSP2,PRKACB
hsa00860	Porphyrin and chlorophyll metabolism	4	1.98E-02	FECH,BLVRB,HMBS,ALAS2
hsa00100	Steroid biosynthesis	3	2.06E-02	SOAT1,CYP2R1,C5orf4
hsa04720	Long-term potentiation	5	2.19E-02	NRAS,RAP1A,RAP1B,RAPGEF3,PRKACB
hsa04120	Ubiquitin mediated proteolysis	7	2.29E-02	CDC34,CDC27,BIRC3,UBE2W,SKP1,UBE2E3,CUL3
hsa05200	Pathways in cancer	11	2.52E-02	IL6,BCL2L1,HIF1A,NRAS,PTEN,BIRC3,IL8,ITGB1,DAPK1,SLC2A1,PTGS2

### Correlation among three disease states

A strong overlap of 191 genes was identified among genes that differentially expressed across CAD, T2D, and T2D + CAD groups when compared with the control group.

Additionally, pairwise Spearman’s correlation coefficient was calculated to assess the correlation among three disease states using the differentially expressed genes in each disease. The results showed a significant correlation among CAD, T2D, and T2D + CAD (*p* <0.0001), suggesting the possibility of shared pathophysiology of the diseases. The correlation between T2D and T2D + CAD was the most significant in the peripheral blood gene expression profiles (Spearman’s rho = 0.6757, *p* <0.0001).

For those overlapping genes across CAD, T2D, and T2D + CAD, they are mainly enriched in GO terms of viral infectious cycle (GO:0019058, p = 0.00014), anti-apoptosis (GO:0006916, p = 0.00017), endocrine pancreas development (GO:0031018, p = 0.0004), innate immune response (GO:0045087, *p* = 0.01), and blood coagulation (GO:0007596, p = 0.03), etc. These common genes were mainly involved in pathways of Ribosome (*p* = 2.77E-06), Focal adhesion (*p* = 0.03), Apoptosis (*p* = 0.03), Small cell lung cancer (p = 0.03), RIG-I-like receptor signaling pathway (*p* = 0.03), Mineral absorption (*p* = 0.04), Leukocyte transendothelial migration (*p* = 0.04), etc (Table 4).

**Table 4:** The significantly enriched pathways for the common differentially expressed genes in T2D, CAD, and T2D + CAD

KEGG ID	KEGG term	Count	FDR	Genes
hsa03010	Ribosome	8	0.00	RPS3A,UBA52,RSL24D1,RPL7,RPL39,RPL21,RPS24,RPS12
hsa04510	Focal adhesion	6	0.03	PTEN,BIRC3,RAP1B,ARHGAP5,ITGB1,MYL12A
hsa04210	Apoptosis	4	0.03	BCL2L1,BIRC3,IRAK3,PRKACB
hsa05222	Small cell lung cancer	4	0.03	BCL2L1,PTEN,BIRC3,ITGB1
hsa04622	RIG-I-like receptor signaling pathway	4	0.03	TBK1,ATG5,TANK,IL8
hsa04978	Mineral absorption	3	0.04	ATP2B1,TRPM7,ATP1B3
hsa04670	Leukocyte transendothelial migration	4	0.04	RAP1B,ARHGAP5,ITGB1,MYL12A
hsa04961	Endocrine and other factor-regulated calcium reabsorption	3	0.04	ATP2B1,ATP1B3,PRKACB
hsa04114	Oocyte meiosis	4	0.04	CDC27,SLK,SMC3,PRKACB
hsa05144	Malaria	3	0.04	HBD,GYPC,IL8
hsa05131	Shigellosis	3	0.05	ATG5,IL8,ITGB1

### Disease-specific PPI network

**Fig. 1:**
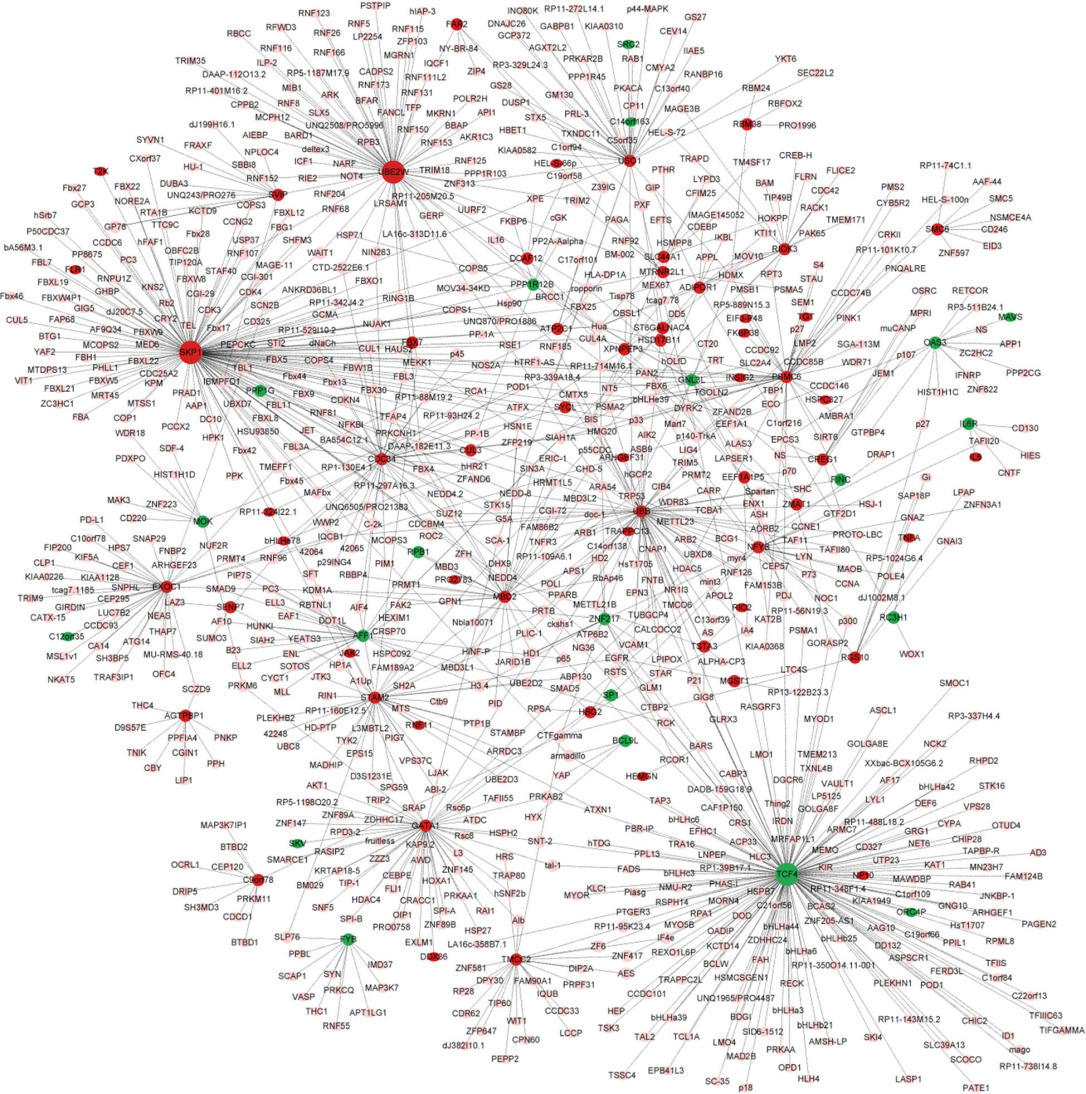
The T2D-specific PPI networks of 64 dysregulated genes. *Red nodes* indicate up-regulated genes in T2D, *blue nodes* indicate down-regulated genes in T2D. *Pink nodes* indicate genes interacting with the differentially expressed genes, and larger icons indicate hub proteins

**Fig. 2:**
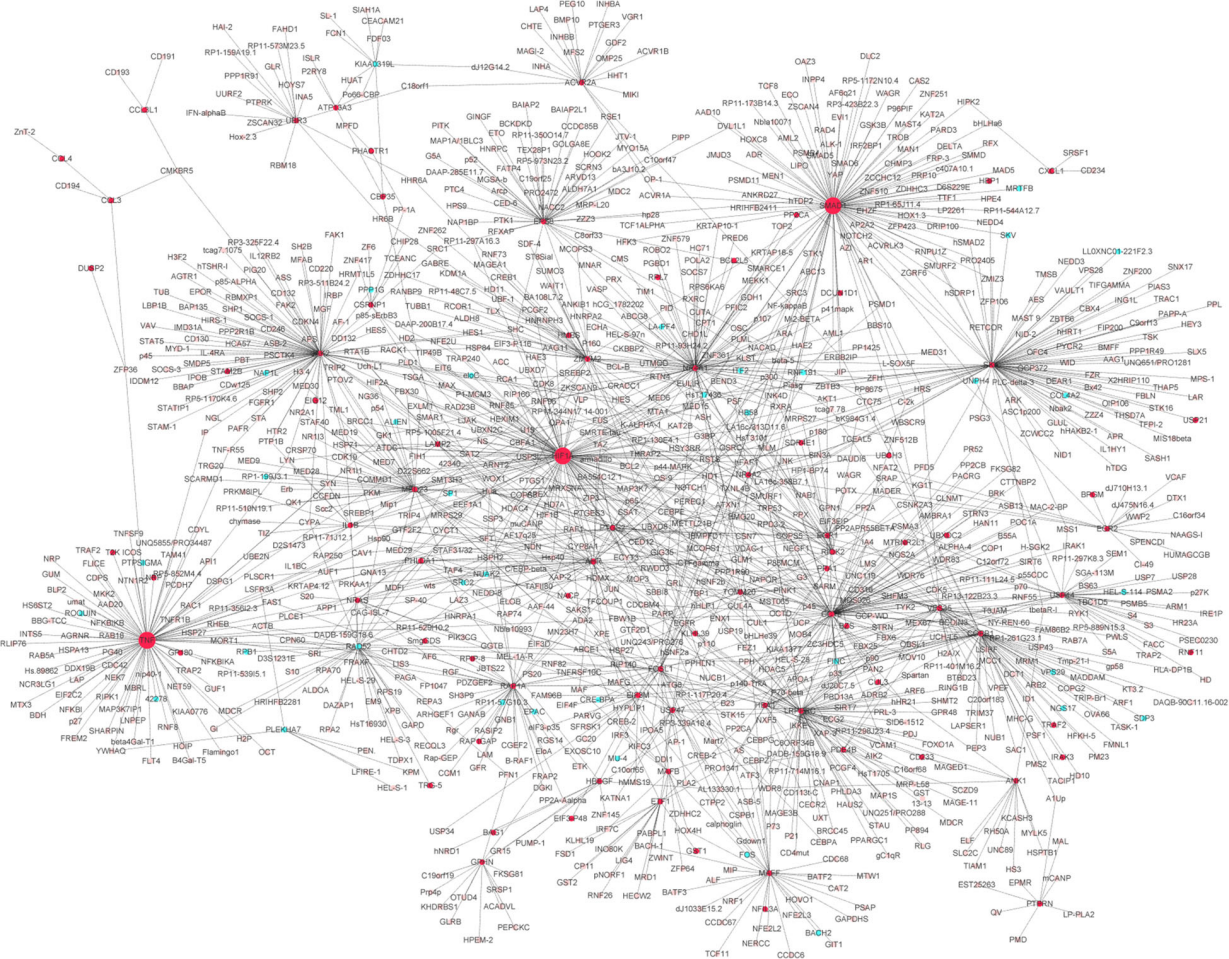
The CAD-specific PPI networks of 69 dysregulated genes. *Red nodes* indicate up-regulated genes in CAD, and *blue nodes* indicate down-regulated genes in CAD. *Pink nodes* indicate genes interacting with the differentially expressed genes, and larger icons indicate hub proteins

The pathology of T2D with CAD shares much in common with that of CAD and T2D. We selected 64 genes that were significantly differentially expressed in T2D or T2D with CAD, but were not significantly differentially expressed in CAD to construct T2D-specific PPI networks. The information of 7 genes wasn’t available in BioGRID database. When removing duplicated edges, self-loops nodes, colocalization edges, finally 47 genes were involved in T2D-specific PPI networks, including 1216 nodes and 1579 edges. The significant hub proteins contained TCF4 (Degree = 169), SKP1 (Degree = 164) and UBE2W (Degree = 75) (Fig. 1), suggesting their important role in the development of T2D. To construct CAD-specific PPI networks, we selected 69 genes that were significantly differentially expressed in CAD or T2D with CAD, but were not significantly differentially expressed in T2D. The information of 9 genes wasn’t available in BioGRID database. When removing duplicated edges, self-loops nodes, colocalization edges, finally 54 genes were involved in T2D-specific PPI networks, including 943 nodes and 1085 edges. The significant hub proteins contained HIF1A (Degree = 124), SMAD1 (Degree = 112) and SKIL (Degree = 94) (Fig. 2), suggesting their important role in the development of CAD.

### The verification of gene expression in GSE23561

**Fig. 3:**
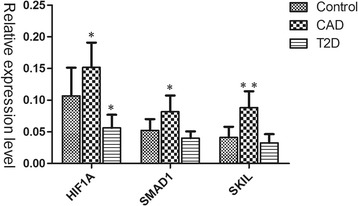
The verification of mRNA expression of HIF1A, SMAD1, and SKIL in patients with CAD or T2D via GSE23561

, HIF1A, SMAD1, and SKIL, specifically differentially expressed in CAD, and also hub genes in the CAD-specific PPI networks, was selected to confirm the above results. With the provided expression data from GSE23561, HIF1A, SMAD1, and SKIL were all significantly up-regulated in CAD, and the expression of SMAD1 and SKIL remained unchangeable in T2D as compared with normal control, which was consistent with our results. Differently, HIF1A was both differentially expressed in CAD and T2D, but with opposite trends (Fig. 3).

## Discussion

In this study, we used RNA-seq to identify unique peripheral blood gene expression signatures of T2D, CAD, and coexisting condition. Previous literature have revealed that there is an intrinsic interplay between T2D and CAD, while the detailed mechanism remains unclear. Toward this end, we compared the gene expression profiles of T2D, CAD and coexisting condition to show the association in-between them, and tried to explain the shared pathophysiology.

Here, PBMCs are usually selected to monitor posttranslational modifications relevant to many diseases [24], updating the underlying molecular events of diseases. Furthermore, the feature of minimal invasion makes differentially expressed genes in PBMCs suitable as predictive biomarkers in clinical studies. Some studies that analyzed gene expression profile in peripheral blood cell of CAD or T2D have already been performed [25–27]. There were common features and characteristic differences between the current study and previous studies. T2D and CAD both manifest disordered coagulation system, local inflammatory process, or lipid-related disorder. The shared pathophysiology between T2D and CAD may be explained by the common genetic variant, such as CDKN2A/2B, ADIPOR1 [28] and TCF7L2 variants [29]. In our study, many differentially expressed genes from the individual comparisons of T2D, CAD and coexisting condition to control was also found to be overlapped among the three disorders, suggesting shared pathophysiology. Also Spearman’s test for gene expression correlation revealed the significant correlation of the three disorders, among which the correlation between T2D and T2D + CAD was the most significant.

The overlapping genes were mainly enriched in GO terms of viral infectious cycle, anti-apoptosis, endocrine pancreas development, innate immune response, and blood coagulation, etc. Those overlapping genes were mainly involved in pathways of Ribosome (RPS3A, UBA52, RSL24D1, RPL7, RPL39, RPL21, RPS24, and RPS12). Ribosomal proteins are involved in cell growth and proliferation, differentiation and apoptosis. Ribosome biogenesis disruption could activate the p53 signaling pathway, resulting in cell cycle arrest and apoptosis. Moreover, it has been correlated to clinical manifestations in pathological states, such as cardiovascular diseases and metabolic disorders. RpL17 inhibited vascular smooth muscle cell growth, and limits carotid intima thickening in mice [30]. Animal study has shown that ribosomal protein S6kinase4 plays a crucial role in pancreatic β-cell function and glucose homeostasis [31]. Ribosomes dysfunction may be applied in the early diagnosis of chronic diseases, such as cancer and cardiovascular diseases.

Besides the common signatures among T2D, CAD and coexisting condition, we also analyzed the gene sets which functioned specially in the development of T2D or CAD. In the T2D-specific PPI networks, the significant hub proteins were TCF4 (Degree = 169), SKP1 (Degree = 164) and UBE2W (Degree = 75). TCF4, also named transcription factor 7-like 2 (TCF7L2), encodes a transcription factor involved in the Wnt signaling pathway. TCF4 stimulates the proliferation of pancreatic β-cells, regulates embryonic development of the pancreatic mass, and induces the production of the insulinotropic hormone glucagon-like peptide-1 (GLP-1) in intestinal endocrine cells [32]. It plays a critical role in blood glucose homeostasis. Recently, numerous studies have demonstrated an association between TCF7L2 genotype and T2D [33–37]. In a meta-analysis by Wang J et al., the TCF7L2 rs7903146 polymorphism was found to be associated with increased T2D risk in the Chinese population [38]. There were also evidences for a strong interplay between TCF7L2 polymorphisms and CAD [29, 39]. In another study on nine hundred subjects referred for cardiac catheterization for CAD diagnosis by Sousa AG et al., a significant association was identified between the TCF7L2 rs7903146 polymorphism and the prevalence and severity of CAD [40].

Interestingly, differentially expressed genes between T2D and normal controls were significantly enriched in Parkinson’s disease, suggesting that a shared pathophysiology of Parkinson’s disease and T2D. The link has been confirmed in several epidemiological studies [41, 42]. The mechanism behind this association may be that there was a reciprocal regulation between insulin and dopamine [43].

In case of CAD-specific PPI networks, the significant hub proteins were HIF1A, SMAD1 and SKIL. In the provided expression data from GSE23561, we found that SMAD1 and SKIL were specifically up-regulated in CAD with no change in T2D, while HIF1A was both differentially expressed in CAD and T2D, but with opposite trends. In CAD patients, oxygen supply was limited as a result of reduced blood flow brought about by atherosclerotic plaque formation and inflammatory processes taking place within the vascular endothelium [44]. As a consequence of oxidative stress in hypoxic conditions, hypoxia-inducible factor-1 alpha (HIF-1α) was produced to involve in adaptive and repair mechanisms. HIF-1α is a transcriptional factor encoded by the HIF1A gene, functioning in the preservation of oxygen homeostasis. HIF-1α may participate in the occurrence and progression of CAD through activating various genes such as VEGF, HO-1, and ET-1. HIF-1α mRNA was found to be markedly up-regulated in both monocytes and lymphocytes of CAD patients than that of controls, and the expression level of HIF-1α was highly correlated with severity of atherosclerosis and higher level of collateral score [45, 46]. In addition, other studies have investigated the correlation between HIF1A polymorphism and CAD. A recent study showed that HIF-1α polymorphisms (rs11549465 and rs11549467) were associated with clinical type and formation of coronary collaterals [47]. The rs2057482 SNP of HIF1A was showed to be associated with increased susceptibility to premature CAD, which may be applied in clinical diagnostics as a susceptibility marker of premature CAD [48].

However, there were some evidences showing that the HIF1A was crucial for first phase insulin secretion and glucose homeostasis. Nagy et al. revealed that polymorphism (g.C45035TSNP, rs11549465) of HIF1A were associated with T1D and T2D in a Caucasian population [49]. Cheng et al. identified that HIF1A could play an important role in β cell function via binding to ARNT promoter in a mouse with β cell–specific Hif1a disruption [50]. Besides, hyperglycaemia appeared to down-regulated HIF-1α mRNA expression in ischaemic myocardium, and inhibited the defective response of HIF-1α to ischaemia [51, 52], explaining a positive association between hyperglycaemia at the time of the event and subsequent mortality from myocardial infarction. The dual role of HIF1A connects the pathogenesis of T2D with that of CAD. For this study, we determined the up-regulation of HIF1A in CAD, but didn’t detect the expression of down-regulation of HIF1A in T2D in RNA-seq results. The limited sample may explain the inconsistency between RNA-seq results and that of published studies. Further large-sample studies are needed to confirm these results.

In our study, SMAD1 and SKIL were both significantly up-regulated in CAD, remained unchangeable in T2D, which were involved in SMAD pathway. SMAD1 polymorphism was reported to be associated with sudden cardiac arrest in CAD patients [53]. In a transgenic mice model with cardiac-specific overexpression of smad1, Masaki M found that transgenic mice had significantly smaller myocardial infarctions and fewer apoptotic deaths of cardiomyocytes after ischemia-reperfusion (I/R) injury, suggesting a role of SMAD1 in cardioprotection against I/R injury [54]. SKIL, a component of the SMAD pathway, was showed to be involved in cardiac fibrosis [55, 56]. There remained no reports on the association with CAD.

## Conclusions

In summary, our data showed that the gene expression profile of T2D, CAD, and coexisting condition were all distinguishable from controls, and displayed common and specific gene expression pattern in each disordered state. To note, viral infectious cycle, anti-apoptosis, endocrine pancreas development, innate immune response and blood coagulation were common biological processes among the three conditions. This study provides some evidences in the transcript level to show the association of T2D, CAD and coexisting condition. For this study, the number of sample for RNA-seq was small, which is a limitation of this study, so studies of large sample size need to be conducted to confirm this conclusion.

## Additional file

Additional file 1: Table S1. Demographic data and medication of the study population. (XLSX 10 kb)

## Abbreviations

BioGRID: Biological general repository for interaction datasets
CAD: Coronary artery disease
GEO: Gene expression omnibus
GO: Gene ontology
KEGG: Kyoto encyclopedia of genes and genomes
PPI: Protein-protein interaction
RPKM: Reads per kilobase of exon per million mapped reads
T2D: type 2 diabetes

## References

[1] Frosteg XJ (2013). rd: immune mechanisms in atherosclerosis, especially in diabetes type 2. Front Endocrinol.

[2] Gong F, Wu J, Zhou P, Zhang M, Liu J, Liu Y, Lu X, Liu Z (2016). Interleukin-22 might Act as a double-edged sword in type 2 diabetes and coronary artery disease. Mediators Inflamm.

[3] Warner D, Mansfield M, Grant PJ (2001). Coagulation factor XIII levels in UK Asian subjects with type 2 diabetes mellitus and coronary artery disease. Thromb Haemost.

[4] Kannel WB, McGee DL (1979). Diabetes and glucose tolerance as risk factors for cardiovascular disease: the Framingham study. Diabetes Care.

[5] Wilson PW, Kannel WB (2002). Obesity, diabetes, and risk of cardiovascular disease in the elderly. Am J Geriatr Cardiol.

[6] Sanchez-Recalde A, Carlos Kaski J (2001). [Diabetes mellitus, inflammation and coronary atherosclerosis: current and future perspectives]. Rev Esp Cardiol.

[7] Wu C, Gong Y, Yuan J, Gong H, Zou Y, Ge J (2012). Identification of shared genetic susceptibility locus for coronary artery disease, type 2 diabetes and obesity: a meta-analysis of genome-wide studies. Cardiovasc Diabetol.

[8] Reimers M, Carey VJ (2006). [8] bioconductor: an open source framework for bioinformatics and computational biology. Methods Enzymol.

[9] Aziz H, Zaas A, Ginsburg GS (2007). Peripheral blood gene expression profiling for cardiovascular disease assessment. Genomic Med.

[10] Braakhuis BJ, Graveland AP, Dijk F, Ylstra B, van Wieringen WN, Leemans CR, Brakenhoff RH (2013). Expression signature in peripheral blood cells for molecular diagnosis of head and neck squamous cell carcinoma. Oral Dis.

[11] Luque MC, Santos CC, Mairena EC, Wilkinson P, Boucher G, Segurado AC, Fonseca LA, Sabino E, Kalil JE, Cunha-Neto E (2014). Gene expression profile in long-term non progressor HIV infected patients: in search of potential resistance factors. Mol Immunol.

[12] Xu Y, Xu Q, Yang L, Liu F, Ye X, Wu F, Ni S, Tan C, Cai G, Meng X (2013). Gene expression analysis of peripheral blood cells reveals toll-like receptor pathway deregulation in colorectal cancer. PLoS One.

[13] Kitajima S, Iwata Y, Furuichi K, Sagara A, Shinozaki Y, Toyama T, Sakai N, Shimizu M, Sakurai T, Kaneko S, Wada T. Messenger RNA expression profile of sleep-related genes in peripheral blood cells in patients with chronic kidney disease. Clin Exp Nephrol. 2016;20(2):218–25. 10.1007/s10157-015-1150-y 26388507

[14] Wingrove JA, Daniels SE, Sehnert AJ, Tingley W, Elashoff MR, Rosenberg S, Buellesfeld L, Grube E, Newby LK, Ginsburg GS, Kraus WE (2008). Correlation of peripheral-blood gene expression with the extent of coronary artery stenosis. Circ Cardiovasc Genet.

[15] Alberti KG, Zimmet PZ (1998). Definition, diagnosis and classification of diabetes mellitus and its complications. Part 1: diagnosis and classification of diabetes mellitus provisional report of a WHO consultation. Diabet Med.

[16] Min JK, Shaw LJ (2008). Noninvasive diagnostic and prognostic assessment of individuals with suspected coronary artery disease: coronary computed tomographic angiography perspective. Circ Cardiovasc Imaging.

[17] Trapnell C, Pachter L, Salzberg SL (2009). TopHat: discovering splice junctions with RNA-Seq. Bioinformatics.

[18] Trapnell C, Roberts A, Goff L, Pertea G, Kim D, Kelley DR, Pimentel H, Salzberg SL, Rinn JL, Pachter L (2012). Differential gene and transcript expression analysis of RNA-seq experiments with TopHat and Cufflinks. Nat Protoc.

[19] Tabas-Madrid D, Nogales-Cadenas R, Pascual-Montano A (2012). GeneCodis3: a non-redundant and modular enrichment analysis tool for functional genomics. Nucleic Acids Res.

[20] Giot L, Bader JS, Brouwer C, Chaudhuri A, Kuang B, Li Y, Hao Y, Ooi C, Godwin B, Vitols E (2003). A protein interaction map of drosophila melanogaster. Science.

[21] Li S, Armstrong CM, Bertin N, Ge H, Milstein S, Boxem M, Vidalain P-O, Han J-DJ, Chesneau A, Hao T (2004). A map of the interactome network of the metazoan C. elegans. Science.

[22] Shannon P, Markiel A, Ozier O, Baliga NS, Wang JT, Ramage D, Amin N, Schwikowski B, Ideker T (2003). Cytoscape: a software environment for integrated models of biomolecular interaction networks. Genome Res.

[23] Barrett T, Wilhite SE, Ledoux P, Evangelista C, Kim IF, Tomashevsky M, Marshall KA, Phillippy KH, Sherman PM, Holko M (2013). NCBI GEO: archive for functional genomics data sets—update. Nucleic Acids Res.

[24] Koncarevic S, Lossner C, Kuhn K, Prinz T, Pike I, Zucht HD (2014). In-depth profiling of the peripheral blood mononuclear cells proteome for clinical blood proteomics. Int J Proteomics.

[25] Manoel-Caetano FS, Xavier DJ, Evangelista AF, Takahashi P, Collares CV, Puthier D, Foss-Freitas MC, Foss MC, Donadi EA, Passos GA (2012). Gene expression profiles displayed by peripheral blood mononuclear cells from patients with type 2 diabetes mellitus focusing on biological processes implicated on the pathogenesis of the disease. Gene.

[26] Mao J, Ai J, Zhou X, Shenwu M, Jr OM, Blue M, Washington JT, Wang X, Deng Y (2011). Transcriptomic profiles of peripheral white blood cells in type II diabetes and racial differences in expression profiles. BMC Genomics.

[27] Sinnaeve PR, Donahue MP, Grass P, Seo D, Vonderscher J, Chibout SD, Kraus WE, Jr SM, Nelson C, Ginsburg GS (2009). Gene expression patterns in peripheral blood correlate with the extent of coronary artery disease. PLoS One.

[28] Jin Z, Pu L, Sun L, Chen W, Nan N, Li H, Zhu H, Yang X, Wang N, Hui J (2014). Identification of susceptibility variants in ADIPOR1 gene associated with type 2 diabetes, coronary artery disease and the comorbidity of type 2 diabetes and coronary artery disease. PLoS One.

[29] Sousa AG, Selvatici L, Krieger JE, Pereira AC (2011). Association between genetics of diabetes, coronary artery disease, and macrovascular complications: exploring a common ground hypothesis. Rev Diabet Stud.

[30] Smolock EM, Korshunov VA, Glazko G, Qiu X, Gerloff J, Berk BC (2012). Ribosomal protein L17, RpL17, is an inhibitor of vascular smooth muscle growth and carotid intima formation. Circulation.

[31] Ruvinsky I, Sharon N, Lerer T, Cohen H, Stolovich-Rain M, Nir T, Dor Y, Zisman P, Meyuhas O (2005). Ribosomal protein S6 phosphorylation is a determinant of cell size and glucose homeostasis. Genes Dev.

[32] Jin T, Liu L (2008). The Wnt signaling pathway effector TCF7L2 and type 2 diabetes mellitus. Mol Endocrinol.

[33] Assmann TS, Duarte GC, Rheinheimer J, Cruz LA, Canani LH, Crispim D (2014). The TCF7L2 rs7903146 (C/T) polymorphism is associated with risk to type 2 diabetes mellitus in Southern-Brazil. Arq Bras Endocrinol Metabol.

[34] Potatoes and neural tube defects. Food Cosmet Toxicol 1973, 11:1134-1135. 4593594

[35] Sgariglia F, Pedrini E, Bradfield JP, Bhatti TR, D’Adamo P, Dormans JP, Gunawardena AT, Hakonarson H, Hecht JT, Sangiorgi L (2015). The type 2 diabetes associated rs7903146 T allele within TCF7L2 is significantly under-represented in hereditary multiple exostoses: insights into pathogenesis. Bone.

[36] Daniele G, Gaggini M, Comassi M, Bianchi C, Basta G, Dardano A, Miccoli R, Mari A, Gastaldelli A, Del Prato S (2015). Glucose metabolism in high-risk subjects for type 2 diabetes carrying the rs7903146 TCF7L2 gene variant. J Clin Endocrinol Metab.

[37] Drake I, Wallstrom P, Hindy G, Ericson U, Gullberg B, Bjartell A, Sonestedt E, Orho-Melander M, Wirfalt E (2014). TCF7L2 type 2 diabetes risk variant, lifestyle factors, and incidence of prostate cancer. Prostate.

[38] Wang J, Hu F, Feng T, Zhao J, Yin L, Li L, Wang Y, Wang Q, Hu D (2013). Meta-analysis of associations between TCF7L2 polymorphisms and risk of type 2 diabetes mellitus in the Chinese population. BMC Med Genet.

[39] Muendlein A, Saely CH, Geller-Rhomberg S, Sonderegger G, Rein P, Winder T, Beer S, Vonbank A, Drexel H (2011). Single nucleotide polymorphisms of TCF7L2 are linked to diabetic coronary atherosclerosis. PLoS One.

[40] Sousa AG, Lopes NH, Hueb WA, Krieger JE, Pereira AC (2011). Genetic variants of diabetes risk and incident cardiovascular events in chronic coronary artery disease. PLoS One.

[41] Hu G, Jousilahti P, Bidel S, Antikainen R, Tuomilehto J (2007). Type 2 diabetes and the risk of Parkinson’s disease. Diabetes Care.

[42] D’Amelio M, Ragonese P, Callari G, Di Benedetto N, Palmeri B, Terruso V, Salemi G, Famoso G, Aridon P, Savettieri G (2009). Diabetes preceding Parkinson’s disease onset. A case-control study. Parkinsonism Relat Disord.

[43] Lima MM, Targa AD, Noseda AC, Rodrigues LS, Delattre AM, dos Santos FV, Fortes MH, Maturana MJ, Ferraz AC (2014). Does Parkinson’s disease and type-2 diabetes mellitus present common pathophysiological mechanisms and treatments?. CNS Neurol Disord Drug Targets.

[44] Shah PK (2003). Pathophysiology of plaque rupture and the concept of plaque stabilization. Cardiol Clin.

[45] Chen SM, Li YG, Wang DM, Zhang GH, Tan CJ (2009). Expression of heme oxygenase-1, hypoxia inducible factor-1alpha, and ubiquitin in peripheral inflammatory cells from patients with coronary heart disease. Clin Chem Lab Med.

[46] Chen SM, Li YG, Zhang HX, Zhang GH, Long JR, Tan CJ, Wang DM, Fang XY, Mai RQ (2008). Hypoxia-inducible factor-1alpha induces the coronary collaterals for coronary artery disease. Coron Artery Dis.

[47] Liu Q, Liang Y, Zou P, Ni WX, Li YG, Chen SM (2013). Hypoxia-inducible factor-1alpha polymorphisms link to coronary artery collateral development and clinical presentation of coronary artery disease. Biomed Pap Med Fac Univ Palacky Olomouc Czech Repub.

[48] Lopez-Reyes A, Rodriguez-Perez JM, Fernandez-Torres J, Martinez-Rodriguez N, Perez-Hernandez N, Fuentes-Gomez AJ, Aguilar-Gonzalez CA, Alvarez-Leon E, Posadas-Romero C, Villarreal-Molina T (2014). The HIF1A rs2057482 polymorphism is associated with risk of developing premature coronary artery disease and with some metabolic and cardiovascular risk factors. The genetics of atherosclerotic disease (GEA) Mexican study. Exp Mol Pathol.

[49] Nagy G, Kovacs-Nagy R, Kereszturi E, Somogyi A, Szekely A, Nemeth N, Hosszufalusi N, Panczel P, Ronai Z, Sasvari-Szekely M (2009). Association of hypoxia inducible factor-1 alpha gene polymorphism with both type 1 and type 2 diabetes in a Caucasian (Hungarian) sample. BMC Med Genet.

[50] Cheng K, Ho K, Stokes R, Scott C, Lau SM, Hawthorne WJ, O’Connell PJ, Loudovaris T, Kay TW, Kulkarni RN (2010). Hypoxia-inducible factor-1alpha regulates beta cell function in mouse and human islets. J Clin Invest.

[51] Marfella R, D’Amico M, Di FC, Piegari E, Nappo F, Esposito K, Berrino L, Rossi F, Giugliano D (2002). Myocardial infarction in diabetic rats: role of hyperglycaemia on infarct size and early expression of hypoxia-inducible factor 1. Diabetologia.

[52] Bento CF, Pereira P (2011). Regulation of hypoxia-inducible factor 1 and the loss of the cellular response to hypoxia in diabetes. Diabetologia.

[53] Tseng ZH, Vittinghoff E, Musone SL, Lin F, Whiteman D, Pawlikowska L, Kwok PY, Olgin JE, Aouizerat BE (2009). Association of TGFBR2 polymorphism with risk of sudden cardiac arrest in patients with coronary artery disease. Heart Rhythm.

[54] Masaki M, Izumi M, Oshima Y, Nakaoka Y, Kuroda T, Kimura R, Sugiyama S, Terai K, Kitakaze M, Yamauchi-Takihara K (2005). Smad1 protects cardiomyocytes from ischemia-reperfusion injury. Circulation.

[55] Cunnington RH, Nazari M, Dixon IM (2009). c-Ski, Smurf2, and Arkadia as regulators of TGF-beta signaling: new targets for managing myofibroblast function and cardiac fibrosis. Can J Physiol Pharmacol.

[56] Kishore R, Verma SK, Mackie AR, Vaughan EE, Abramova TV, Aiko I, Krishnamurthy P (2013). Bone marrow progenitor cell therapy-mediated paracrine regulation of cardiac miRNA-155 modulates fibrotic response in diabetic hearts. PLoS One.

